# Pharmacological Treatment of Mild Cognitive Impairment as a Prodromal Syndrome of Alzheimer´s Disease

**DOI:** 10.2174/157015913804999487

**Published:** 2013-01

**Authors:** Tarik Karakaya, Fabian Fußer, Johannes Schröder, Johannes Pantel

**Affiliations:** 1 Department of Psychiatry, Psychosomatic Medicine and Psychotherapy, Goethe-University, Frankfurt am Main, Germany; 2 Department of Psychiatry, Section of Geriatric Psychiatry, University of Heidelberg, Heidelberg, Germany; 3 Institute of General Practice, Geriatric Medicine, Goethe-University, Frankfurt am Main, Germany

**Keywords:** Mild cognitive impairment, Alzheimer’s Dementia, Clinical Trials, Treatment.

## Abstract

Mild cognitive impairment (MCI) is a syndrome which, depending on various neurobiological, psychological and social factors, carries a high risk of developing into dementia. As far as diagnostic uncertainty and the heterogeneous underlying pathophysiological mechanisms are concerned, only limited therapeutic options are currently available. Clinical trials involving a wide range of substances have failed to show efficacy on primary and secondary outcome parameters. Most results reflect not only a lack of effectiveness of drug therapy but also methodological constraints in true prodromal Alzheimer´s disease (AD) based on clinical criteria. Biomarkers may help to identify MCI as a prodromal phase of dementia, so it is important to use them to improve specificity of case selection in future studies. For MCI as a prodromal syndrome of AD, clinical trials with disease modifying drugs that target underlying pathological mechanisms such as amyloid-beta accumulation and neurofibrillary tangle formation may help develop effective treatment options in the future. Alternative pharmacological approaches are currently being evaluated in ongoing phase 1 and phase 2 studies. Nevertheless, a lack of approved pharmacotherapeutic options has led to specific interventions that focus on patient education and life-style related factors receiving increasing attention.

## INTRODUCTION

The term mild cognitive impairment (MCI) is defined as cognitive decline that is above age- and education-adjusted norms but does not fulfil clinical criteria for dementia [1-4] (see also Box **1**). Currently, diagnostic uncertainty commonly persists, even after full diagnostic screening for this hetero- geneous syndrome has occurred. Without any approved pharmacological treatment and only modest evidence for symptomatic treatment efficacy [5] therapeutic options are severely limited. Besides non-pharmacological interventions (e.g. cognitive stimulation), a few pharmacological treatment approaches exist that are mostly based on the assumption that MCI is a transitional pre-dementia stage of Alzheimer´s Disease (AD) with positive biomarkers related to the pathophysiological mechanisms of AD [6].

The criteria for MCI due to AD have very recently been revised by a working group assigned by the National Institute on Aging (NIA) and the Alzheimer’s Association. Besides core clinical criteria, the revised research criteria (e.g. for clinical trials) implement such biomarkers as neuroimaging methods and cerebrospinal fluid evaluation and lead to four levels of diagnostic certainty [7] (see Table **1**). For the highest level of certainty it is assumed that an underlying pathology of AD is very probable. Following this stratification a pharmacological intervention might then target the pathologic processes even at an early clinical stage when cognitive impairments are still slight. Nonetheless, a prospective evaluation of sensitivity, specificity, and the predictive value of the proposed criteria is pertinent before its use can be generally recommended.

Some of the major causes of MCI that have no pathophysiological link to AD are shown in Table **2**. Many of them can be treated effectively and should be identified or ruled out during diagnostic screening procedures.

An appropriate target for pharmacological intervention should be directly linked to patient-relevant outcomes of clinical treatment trials. One aim of the therapy should be to improve subjective, and more importantly objectively measurable, cognitive performance. Another important goal of the therapy is to prevent the progression of cognitive deficits and ultimately to delay the conversion of MCI to clinically manifest dementia. This includes primary and secondary preventive options. It should be noted that prevention of clinical progression or conversion to dementia, or even the stabilization of the cognitive state (possibly including a persistence of low-grade cognitive deficits) should be regarded as successful treatment.

Assuming that most MCI patients, especially patients with amnestic MCI [8], have an underlying pathology of AD, it is logical to investigate whether drug treatment strategies for AD might be effective in the treatment of MCI (e.g. treatment with acetylcholinesterase inhibitors (AChEI) or ginkgo biloba). To date, randomized controlled trials

**Box 1.** General Criteria for MCI (Modified after [3])

● Diagnosis based on clinical judgement
● Report of cognitive decline by the patient and / or an informant
Cognitive impairment in one or more domains (e.g. memory, attention, language, visuospatial skills, perceptual speed or executive functioning).: memory impairment (amnestic MCI) multidomain MCI (either amnestic or non-amnestic) single non-memory domain MCI
● Evidence of decline present in neuropsychological assessment.
● Mainly preserved basic activities of daily living (i.e. no dementia)

(RCTs) have evaluated a variety of substances that have been hypothesized to be useful in AD. These include non-steroidal anti-inflammatory drugs (in particular rofecoxib), statins, antioxidants, insulin and platelet aggregation inhibitors. Some RCTs have examined pharmacological treatment using antidepressants. So far no reliable data are available for Memantine.

## PHARMACOLOGICAL TREATMENT OF MCI

### Acetylcholinesterase-Inhibitors

Various studies have identified the cholinergic deficit as a pathophysiological cause of cognitive deficits in AD. Evidence from early histological postmortem studies [9] is consistent with the reduction in cortical acetylcholinesterase (AChE) activity demonstrated in PET studies [10] and the atrophy of the nucleus basalis of Meynert as revealed using structural MRI [11]. This nucleus in the basal forebrain is the major source of AChE, as well as the origin of cholinergic neurotransmission and projections to cortical brain areas associated with learning and memory, e.g. the hippocampus. Pharmacological treatment in AD (and MCI; for AChEI treatment of other dementias see also [12]) focused on increasing the levels of synaptic acetylcholine via the reversible inhibition of acetylcholinesterase activity (donepezil, galantamine), thus improving cognitive functioning. Rivastigmine additionally inhibits butyrylcholinesterase. Results from RCTs on MCI are available for the three acetylcholinesterase inhibitors (AChEI): donepezil, rivastigmine and galantamine which were systematically reviewed [13, 14].

Petersen and colleagues performed the first large trial for the treatment of MCI with *donepezil* (in combination with vitamin E) [15]. The primary endpoint of the prospective Memory Impairment Study (MIS) was conversion to clinically manifest dementia following treatment with 10 mg donepezil, or 1000 IU of vitamin E. The mean age of the participants was 72 and 55% of them were carriers of at least one apolipoprotein E4 (APOE4) allele. After three years and with a trial completion rate of 70%, 214 participants converted to manifest dementia, corresponding to an annual conversion rate of 16%. Patients treated with donepezil showed only minor improvement in the first observation phase (18 months) of the trial. During the second phase, the gap between the two groups narrowed and no significant differences between the three treatments (donepezil versus vitamin E versus placebo) could be identified. During the 3 years of treatment 23 patients died, of which 10 belonged to the donepezil group, 7 to the placebo group and 6 to the vitamin E group. 76% of the patients who converted to dementia were APOE4 allele carriers. 24 months after starting treatment, donepezil could be observed to have a positive effect within the APOE4 group, but on completion of the trial, this effect had significantly decreased.

While in the Petersen study, the secondary preventive effect of donepezil on MCI was investigated, Salloway *et al*. [16] focused more on short-term symptomatic effects. In this 24-week randomized double-blind placebo-controlled trial, cognitive functioning using the ADAS-Cog and various declarative memory tasks were investigated in 269 patients. In addition, functional status was assessed. In the donepezil group an improvement in ADAS-Cog scores, declarative memory and the self-assessment of study participants was observed. An interesting point is that the rate of side-effects was higher in MCI patients (88% donepezil treatment, 73% placebo treatment) than in former trials involving AD patients.

The results of the so-called InDDEx trial with *rivastigmine* were reported by Feldman *et al*. [17]. Similar to the above-cited trial by Petersen [15] the primary endpoint was the time to conversion to clinically manifest dementia. Another outcome variable was cognitive performance and variations in it during treatment. 1018 participants were included in the randomized double-blind, placebo-controlled trial, and half of them were treated with 3-12 mg of rivastigmine per day. 17.3% of the patients in the rivastigmine group and 21.4% of patients in the placebo group converted to dementia within 4 years. There were no significant differences in conversion rates and cognitive performance parameters between active treatment and placebo groups. The number of serious adverse events was relatively high at 27.9% in the rivastigmine group and 30.5% in the placebo group, with most side effects related to the side effects of the substance (nausea, vomiting, diarrhea, dizziness). Therefore, treatment with rivastigmine could not be recommended on the basis of this trial.

The results of 2 large trials with *galantamine* (GAL-INT-11, GAL-INT-18) were also disappointing [5]. A sample of 2048 patients with MCI participated and the patients were randomized to two groups, one of which was treated with galantamine (16-24 mg / day) and the other with a placebo. Over a period of 24 months the conversion rate to dementia and cognitive functioning were investigated using various measures. After completion of the studies, there were no significant differences in the conversion rates between the treatment and non-treatment groups (study I: 22.9% versus 22.6%, study II: 25.4% versus 31.2%). Individual cognitive performance variables (Digit Symbol Substitution Test) were improved in the treatment group in Study I after 12 months and in Study II after 24 months. At 19%, adverse events occurred in both groups. However, the mortality of subjects undergoing galantamine treatment increased significantly compared to the placebo group (1.4% versus 0.3%). Furthermore a significantly lower hippocampal atrophy rate - measured using MRI - was observed in the verum group. Unfortunately, this dataset has not yet been published by Janssen.

According to Winblad *et al*. [5] the results of GAL-INT-11 and GAL-INT-18 showed a slight but significantly increased mortality risk during treatment with galantamine, suggesting that pharmacological treatment of MCI patients should be undertaken carefully.

In a recent 2-year, double-blinded, placebo-controlled study, 232 MCI patients were administered 16 mg of galantamine combined with 20 mg of memantine, galantamine only, or a placebo [18]. Progression to dementia and cognitive changes were assessed using the ADAS-Cog. Despite some methodological issues (early discontinuation due to safety concerns and different treatment durations), the amnestic MCI subgroup in the treatment arm combining galantamine and memantine demonstrated a significant positive effect on cognition. Discontinuation of galantamine, but not memantine led to a decline in cognitive functioning. It therefore seems reasonable to assume that AChEI may improve cognition among amnestic MCI patients by targeting the assumed underlying AD pathology.

### Memantine

Despite the abovementioned combination with AChEI for the treatment of MCI patients with memantine, no reliable data from an RCT is yet available on the influence of memantine. It even seems likely, that memantine alone shows no efficacy in the treatment of mild AD [19].

### Ginkgo Biloba

Ginkgo Biloba has been used to improve cognitive functioning in older people for many years. Recent guidelines for the biological treatment of AD and other dementias stress that the standardized plant extract (EGb 761) has a modest effect on the cognitive function of AD patients that is comparable to that of AChEI or memantine, and it has fewer side effects [20]. EGb 761 contains flavone glycosides (quercetin, kaempferol, isorhamnetin) and terpene lactones (ginkgolides and bilobalide) and has antioxidant and free radical-scavenging activities [21]. Translational studies have also shown its potential to inhibit amyloid-aggregation and consecutive mitochondrion-initiated apoptosis, and indicated that direct neuronal damage may actually be mediated by the amyloid toxicity [22].

The largest trial investigating Ginkgo Biloba’s impact on the primary and secondary prevention of dementia was also of the highest methodological quality. The results of the trial were published by DeKosky and colleagues in 2008 [23]. The Ginkgo Evaluation of Memory (GEM Study) study was conducted in the years 2000-2008 as a randomized, double-blind placebo-controlled multicenter trial in the United States. 3069 participants were included with an average age of 75, the majority of individuals (n=2587) had no cognitive impairment, and 15.70 % (n=482) suffered from MCI according to Petersen’s criteria [1, 2].

After completion of the observation period (median 6.1 years), most patients (60.3%) were taking medication regularly. The rate of dementia was 3.3 per 100 person-years among participants who received Ginkgo Biloba and 2.9 per 100 person-years in the placebo group. Hence, no significant effect of Ginkgo Biloba on the incidence of dementia during the observation period could be demonstrated. A separate analysis of participants with MCI showed an identical result. The authors concluded that the daily intake of 2 x 120 mg Ginkgo Biloba does not provide any benefit to healthy older people, or patients with MCI.

According to the Cochrane Library, evidence that Ginkgo Biloba has predictable and clinically significant benefits for people with dementia or cognitive impairment is inconsistent and unreliable [24].

### Anti-inflammatory Drugs (NSAIDs)

Several large epidemiological studies have demonstrated a negative association between the use of non-steroidal anti-inflammatory drugs and the development of AD [25]. This has led to an investigation into the secondary preventive effect of these substances in people with MCI. So far, only one large multicenter study on the efficacy of the COX-II inhibitor *rofecoxib *(25 mg / day taken by people with MCI until the onset of dementia) has been completed. A sample of 1457 subjects with MCI participated, half of whom were given rofecoxib and the other half a placebo over a period of up to 4 years [26, 27]. Since the conversion rate to dementia over the course of the study turned out to be much lower than expected (6.4% in the rofecoxib group versus 4.5% in the placebo group; expected rate of 10-15% per year), the study was discontinued. At this time there were no significant differences between the two groups in terms of either conversion rates or cognitive parameters. These findings suggest that the administration of NSAIDs for the secondary prevention of dementia in persons with MCI cannot be recommended at this stage.

### Statins

Epidemiological and experimental *in vitro* and *in vivo* studies have indicated a link between cholesterol metabolism and the development of AD. It is assumed that there is an influence of cholesterol on the formation and accumulation of amyloid-beta (Aβ) [28, 29]. In contrast to peripheral cholesterol metabolism, neural cholesterol is not affected by food or dietary factors. The cholesterol level in the human brain is largely derived from in situ synthesis and can be modified through the use of statins. High doses of particularly lipophilic statins (lovastatin, simvastatin) can cross the blood-brain barrier and thus exert an influence on cerebral cholesterol metabolism.

In a 26-week randomized, controlled, double-blind trial, 80 mg *simvastatin* was administered to 44 patients with normal cholesterol levels and 40 patients with AD. A significant reduction in CSF Aβ that correlated with a reduction in "neural" 24 S -hydroxycholesterol and the slower progression of clinical symptoms were observed [30].

To date two large population-based cohort studies of cognitively healthy persons over 60 years of age have provided conflicting results regarding the primary preventive effect of statins on AD [31, 32]. Additionally, two intervention studies investigating the primary prevention of cognitive deficits using statins came to the result that statins reduce cardiovascular and cerebrovascular risk, but show no advantage in cognitive functioning [33, 34]. These results may be due to the methodological limitations of the studies, especially the relatively short study duration and the limited or incomplete measurement of cognitive function. The dose of statins used in the study may also play a role, since only a specific (high) dose crosses the blood-brain barrier and thereby influences the neuronal metabolism.

According to the Cochrane library, there is substantial evidence from RCTs that statins given late in life to individuals at risk of vascular disease do not prevent AD or dementia although biologically it seems feasible that statins could prevent dementia due to their role in cholesterol reduction [35].

RCTs that investigated the effect of statins in the secondary prevention of MCI are currently unavailable. A multicenter study on the daily use of 60 mg simvastatin is currently being undertaken and funded in Germany by the BMBF (Federal Ministry of Education and Research) (see Table **3**).

### Platelet Aggregation Inhibitors (Triflusal)

The effect of the platelet aggregation inhibitor *triflusal* on cognitive parameters (primary endpoint) and conversion to dementia (secondary endpoint) has been studied in patients with amnestic mild cognitive impairment (aMCI). Following the inclusion of 250 participants the study was discontinued due to a very slow recruitment rate [36]. Participants were followed up after an average of 13 months, and the analysis of the data showed a significant reduction in the rate of conversion to dementia, even though the primary goal of the study had not been achieved. These results support the further investigation of the potential effects of platelet aggregation inhibitors. In addition to the antiplatelet effect, triflusal has an anti-inflammatory effect, which may explain a potential secondary preventive effect. Further studies on platelet aggregation inhibitors in people with MCI (e.g. use of aspirin) have not yet been performed.

### Piracetam

Piracetam is a cyclic derivative of gamma-aminobutyric acid (GABA) and is already used for the treatment of brain disorders. According to the Cochrane Library, the limited availability of data means there is no evidence that piracetam improves cognitive performance in those with dementia. The efficacy of piracetam on MCI has been studied in a 12-month multi-centric study [13]. The active treatment in this three-arm study was the administration of either 9600 or 4800 mg piracetam per day. While the study confirmed the relatively good tolerability of piracetam, no significant differences between active treatment and placebo were found on the primary and secondary (cognitive) variables. These findings suggest that currently piracetam should not be recommended for the treatment of MCI.

### Other Substances

In addition to the above-mentioned drugs a number of other substances have been investigated in terms of their effectiveness and benefits for persons with MCI. These include intranasal *insulin* [37], *melatonin* [38], *nicotine patches *[39], Chinese plant extracts [40] and other antioxidants, various nutritional supplements (including vitamins and omega-3 fatty acids), *ampakines*, *testosterone*, *metformin* and *levodopa.*

However, only studies of small sample size and short duration exist. Based on these data, a recommendation for the use of these substances for the treatment of MCI cannot be given.

Though the majority of registered ongoing trials (see: www.clinicaltrials.gov) test cognitive interventions, a few substances are currently in phase II trials: The efficacy of *ladostigil*, a dual acetylcholine-butyrylcholinesterase and brain selective monoamine oxidase (MAO)-A and -B inhibitor [41] is currently being investigated in a 3-year, randomized, controlled, double-blind, multicenter, phase II clinical trial involving 200 patients. Other drugs under investigation for MCI include *levetiracetam*, *atomoxetine*, *pioglitazone*, *insulin*, *human growth hormones *and *immunoglobulins* (see Table **3**).

## CONCLUSION

The results of the cited studies are sobering. Although a number of fairly large randomized controlled trials investigating drug treatment for MCI are available, they could not achieve their defined primary endpoints. Further studies were discontinued for methodological or safety reasons. So there is no evidence-based treatment option available for MCI. However, it should be noted that the lack of evidence of an effect of these substances is not necessarily the same as ineffectiveness. Rather, methodological issues surrounding the planning and implementation of relevant studies may have played a major role in producing these disappointing results. The thorough selection and stratification of participating patients, e.g. by characterizing biomarker profiles in amnestic MCI patients may be crucial in this respect.

Apart from such methodological issues, the pharmacological mechanism of action for the substance under study needs careful consideration. With respect to MCI as a prodromal syndrome of AD, clinical trials with disease modifying drugs targeting underlying pathological mechanisms such as amyloid-beta accumulation and neurofibrillary tangle formation may represent a promising approach in the search for effective treatment options in the future. Unfortunately, during the last couple of years several trials investigating anti-amyloid strategies for the treatment of AD have failed. These failures provide a lesson which should be taken into consideration when implementing future trials of disease-modifying approaches [42].

Patient education should be undertaken cautiously and should take into account that cognitive deficits may remain stable or even remit spontaneously. Patients should pay attention to lifestyle-related factors, such as diet (e.g. Mediterranean diet), physical activity, weight reduction, and an active lifestyle [43, 44]. The advantage of these considerations is that they give the patient an active role in dealing with the disease and this can have a positive influence on overall quality of life.

## Figures and Tables

**Table 1.** Revised Clinical and Research Criteria for MCI Due to AD (Modified after [7])

Diagnostic Category	Biomarker Probability of AD Etiology	Aβ (PET or CSF)	Neuronal Injury (tau, FDG, sMRI)
MCI–criteria	ambiguous	conflicting, indeterminant or untested	conflicting, indeterminant or untested
MCI due to AD - intermediate likelihood	Intermediate	positive	untested
		untested	positive
MCI due to AD - high likelihood	High	positive	positive
MCI due to AD - unlikely	Low	negative	negative

AD: Alzheimer's disease; Aβ: amyloid beta peptide; PET: positron emission tomography; CSF: cerebrospinal fluid; FDG: fluorodeoxyglucose; sMRI: structural magnetic resonance imaging.

**Table 2.** Possible Underlying Diseases for Non AD MCI (Revised after [45])

**I Systemic Diseases**
malnutrition, vitamin deficiencies, endocrinological disorders, collagenosis / vasculitides,
lupus erythematosus (LE), sarcoidosis, Hashimoto encephalitis, polyarteritis nodosa, and others
Other chronic obstructive pulmonary diseases, radiation, hypoxia, hemodialysis

**II Neurological Diseases**
inflammatory diseases, chronic meningoencephalitis (HIV, neurosyphilis,
neuroborreliosis, herpes simplex encephalitis, etc.), autoimmune diseases (including Hashimoto's encephalitis), para-neoplastic syndrome with cerebral involvement (including limbic encephalitis)
vascular diseases, residual states after acute cerebral ischemia or bleeding, hydrocephalus, normal pressure hydrocephalus, brain tumors and metastases, residual states after traumatic brain, genetic disorders

**III Medication**
sedatives, lithium,. narcotics, antihypertensives,
cimetidin, tranquillizer, antidepressants, analgetics

**IV Metabolic dysfunction**
liver failure with cerebral degeneration, chronic renal failure, lipometabolic disorders and others

**V Psychiatric Disorders**
affective disorders, especially depressive disorders, substance abuse and others

**Table 3.** Selected Ongoing Clinical Trials for the Treatment of MCI

Drug	Class / Function	Phase	Recruiting	Duration	n	Multicenter
**Phosiphen** [(+)-phenserine tartrate	Inhibitor of amyloid precursor protein (APP) synthesis	I	Yes	10 days	30	No
**HPP854**	Beta-secretase-1 (BACE) inhibitor	I	Yes	28 days	30	No
**E2609**	Beta-secretase-1 (BACE) inhibitor	I	Not yet	8 days	50	No
**Atomoxetine**	Selective norepinephrine reuptake inhibitor (NRI)	II	Not yet	6 months	40	No
**Ladostigil**	Combined reversible acetylcholinesterase-butyrylcholinesterase inhibitor, and irreversible monoamine oxidase B inhibitor,	II	Yes	36 months	200	Yes
**LY2886721**	Beta-secretase-1 (BACE) inhibitor	II	Yes	26 weeks	129	Yes
**Levetiracetam**	Exact mechanism unknown. Enhancement of glutamatergic excitatory synaptic transmission	II	Yes	2 weeks	144	No
**NewGam 10% IVIG**	Intravenous immunoglobulin (IVIG)	II	Yes	24 months	50	No
**Insulin detemir**	long-acting human insulin analogue	II	Yes	120 days (SL120)	90	Yes
**Curcumin**	Exact mechanism unknown. Antioxidant and anti-inflammatory properties, decrease in Aβ	II	Yes	18 month	132	No
**Simvastatin**	HMG COA reductase inhibitor	IV	Yes	24 months	640	Yes
**Rivastigmine** (Exelon transdermal patch)	Reversible acetylcholinesterase-butyrylcholinesterase inhibitor	N/A	Yes	24 week	120	No

(Reference:www.clinicaltrials.gov; accessed on: June 8th, 2012)
